# The Potential Diagnostic Value of Exosomal Long Noncoding RNAs in Solid Tumors: A Meta-Analysis and Systematic Review

**DOI:** 10.1155/2020/6786875

**Published:** 2020-08-15

**Authors:** Zilong Wu, Zihao Xu, Boyao Yu, Jingtao Zhang, Bentong Yu

**Affiliations:** ^1^The First Clinical Medical College, Nanchang University, Nanchang, Jiangxi, China; ^2^School of Public Health, Nanchang University, Nanchang, Jiangxi, China; ^3^Xiangya School of Medicine, Central South University, Changsha, Hunan 410013, China; ^4^Department of Thoracic Surgery, The First Affiliated Hospital of Nanchang University, Nanchang, Jiangxi, China

## Abstract

**Background:**

Exosomes are defined as small membranous vesicles. After RNA content was discovered in exosomes, they emerged as a novel approach for the treatment and diagnosis of cancer. Long noncoding RNAs (lncRNA), a kind of specific RNA transcript, have been reported to function as tumor growth, metastasis, invasion, and prognosis by regulating the tumor microenvironment in exosomes. This study aims at exploring the potential diagnostic of exosomal lncRNA in solid tumors.

**Methods:**

A meta-analysis conducted from January 2000 to October 2019 identified publications in the English language. We searched all relevant English literature from the Web of Science, EMBASE, and PubMed databases through October 1, 2019. The articles were strictly screened by our criteria and critiqued using the Preferred Reporting Items for Systematic Reviews and Meta-Analyses guidelines.

**Results:**

There were 28 studies with 19 articles (4017 patients) identified, including studies on gastric cancer, laryngeal squamous cell carcinoma, colorectal cancer, cholangiocarcinoma, breast cancer, esophageal squamous cell carcinoma, hepatocellular carcinoma, nonsmall cell lung cancer, and prostate cancer. A meta-analysis showed that the combined value of sensitivity in 29 studies was 0.74 (95% confidence interval [CI], 0.7–0.78), and the combined value of specificity in the studies was 0.81 (95% CI, 0.78–0.83). This suggests the high diagnostic efficacy of liquid exosomes in cancer patients. It is statistically insignificant in terms of sex, ethnicity, and year. The diagnostic power of urinary system tumors was found to be higher than that of digestive system tumors by several subgroup analyses.

**Conclusions:**

We performed a meta-analysis and literature review of 28 studies that included 4017 patients with 10 malignant cancer types. Mechanistically, our study demonstrated that lncRNAs in exosomes could be a promising bioindicator for the diagnosis and prognosis of solid tumors. INPLASY Registration Number: INPLASY202060083.

## 1. Introduction

In the past several decades, cancer therapy has been studied extensively, which has become the most lethal causes of worldwide [1]. During the past century, there has been a dramatic development in cancer treatments, including surgery, radiotherapy, and immunotherapy [2]. However, the overall survival of malignant tumor patients is still unsatisfactory, and the diagnostic efficiency remains lower than desired, especially for advanced tumor patients. With the development of early diagnosis and treatment of cancer, we observed that an ideal biomarker could play a pivotal role [3, 4]. Thus, it is our primary goal to explore a new effective biomarker for early diagnosis, prediction of prognosis, and ideal therapeutic target for cancer patients.

Exosomes are defined as small membranous vesicles, which are 40 to 150 nm in diameter, with specific surface molecular markers such as CD9, CD63, Hsp70, and TSP101 [5, 6]. After RNA content was discovered in exosomes in 2007, they emerged as a novel approach for the treatment and diagnosis of cancer [7]. During the past 2 decades, many researchers have demonstrated that exosomes could contain various kinds of RNAs, including messenger RNAs (mRNA) [8], microRNAs (miRNA) [9], long noncoding RNAs (lncRNA) [10], and circular RNAs (circRNA) [11]. Exosomes in tumor cells have been found to activate specific tumor resistance responses and promote the occurrence and development of tumors [12]. Despite that, a large number of studies have reported that exosomes are involved in angiogenesis and metastasis, drug resistance, immune related, cytokines secretion, apoptosis, cell proliferation, and oncogenic cell transformation [13]. According to an investigation by Mudgapalli et al., exosomes could act as a messenger between the microenvironment and tumor cells that support leukemia growth, inducing oncogenic factors such as c-Myc [14]. In addition, serum-derived exosomes in primary prostate or prostate metastasis increase a high level of PKM2 expression, suggesting that exosomes could be a potential clinical biomarker in prostate cancer [15].

lncRNAs are defined as a type of specific RNA transcript longer than 200 nucleotides, which were once considered unfunctional transcriptional noise [16]. However, it was later found that ncRNAs play a functional role in carcinogenesis, tumor regulation, and gene expression [17]. Furthermore, lncRNA expression always demonstrates poor prognosis in several cancers, including lung cancer [18], osteosarcoma [19], bladder cancer [20], ovarian cancer [21], liver cancer [22], gastric cancer [23], hepatocellular carcinoma [24], and breast cancer [25]. In these studies, there is an obvious connection between high-level expression of lncRNA and worse clinicopathological outcome, such as distant metastasis, tumor size, lymph node metastasis, clinical stage, and drug resistance. For instance, high SNHG11 expression promotes metastasis and proliferation in colorectal cancer by targeting the Hippo pathway [26]. A study by Fang et al. reported that miR-223 could be mediated, as silencing by DLX6-AS1 promotes the progression of bladder cancer through the upregulation of HSP90B1 [27]. In another study, Xu et al. examined lncRNA PVT1, an oncogene that has been found to result in a poor overall survival in esophageal adenocarcinoma though high-level expression [28]. In addition, in our precious research, we demonstrated that the expression of lncRNA SNHG1 was significantly associated with worse clinical outcome [29]. Mechanistically, it has been reported to function as tumor growth, metastasis, invasion, and prognosis by regulating the tumor microenvironment.

Recent studies have shown that biomarkers with tumors are a hot topic, with researchers focusing on the value of ncRNAs, mRNAs, and exosomes in the diagnosis and treatment of cancer. Most of the available literature on these biomarkers was focused mainly on ncRNAs in blood. In addition to these primary data, systematic studies on the prognosis biomarkers are still needed for further exploration. Now that researchers are aware of the significance of ncRNA in exosomes, the function of circRNAs in exosomes has already been reported as a suitable diagnostic biomarker for tumors, especially in hepatocellular carcinoma [30]. However, the diagnostic effectiveness of lncRNAs in exosomes is still not clear.

Therefore, our team is the first to summarize the key role functional lncRNAs play in different types of tumors by performing a meta-analysis. The specificity and sensitivity of exosomal lncRNAs were evaluated to assess their feasibility as biomarkers of cancer diagnosis.

## 2. Materials and Methods

### 2.1. Literature Search

Guided by the Preferred Reporting Items for Systematic Reviews and Meta-Analyses criteria (http://prismastatement.org) (Figure S1, Table S1), we conducted a meta-analysis by searching all relevant English-language literature from the Web of Science, EMBASE, and PubMed databases through October 1, 2019. The literature search according to the Population, Intervention, Comparator, and Outcomes (PICO) framework was performed, and the criteria for study eligibility were established. The population was defined as patients with cancer. The intervention was defined as the study that should provide the expression levels of exosomal lncRNA. The Comparator was the clinical histopathological outcome. Outcomes considered for study included sensitivity, specificity, the diagnostic likelihood ratio negative (DLR-), diagnostic likelihood ratio positive (DLR+) with corresponding 95% confidence intervals (CI). Keywords searched were (((((((((((((((((((((((((((RNA, Long Noncoding[Title/Abstract]) OR Noncoding RNA, Long[Title/Abstract]) OR lncRNA[Title/Abstract]) OR Long ncRNA[Title/Abstract]) OR ncRNA, Long[Title/Abstract]) OR RNA, Long Non-Translated[Title/Abstract]) OR Long Non-Translated RNA[Title/Abstract]) OR Non-Translated RNA, Long[Title/Abstract]) OR RNA, Long Non Translated[Title/Abstract]) OR Long Non-Coding RNA[Title/Abstract]) OR Long Non Coding RNA[Title/Abstract]) OR Non-Coding RNA, Long[Title/Abstract]) OR RNA, Long Non-Coding[Title/Abstract]) OR Long Non-Protein-Coding RNA[Title/Abstract]) OR Long Non-Protein-Coding RNA[Title/Abstract]) OR Long Non Protein Coding RNA[Title/Abstract]) OR Non-Protein-Coding RNA, Long[Title/Abstract]) OR RNA, Long Non-Protein-Coding[Title/Abstract]) OR Long Noncoding RNA[Title/Abstract]) OR RNA, Long Untranslated[Title/Abstract]) OR Long Untranslated RNA[Title/Abstract]) OR Untranslated RNA, Long[Title/Abstract]) OR Long ncRNAs[Title/Abstract]) OR ncRNAs, Long[Title/Abstract]) OR Long Intergenic Non-Protein Coding RNA[Title/Abstract]) OR Long Intergenic Non Protein Coding RNA[Title/Abstract]) OR LincRNAs[Title/Abstract]) OR LINC RNA[Title/Abstract]) AND (((((((((((((((((Neoplasms[Title/Abstract]) OR Neoplasia[Title/Abstract]) OR Neoplasias[Title/Abstract]) OR Neoplasm[Title/Abstract]) OR Tumors[Title/Abstract]) OR Tumor[Title/Abstract]) OR Cancer[Title/Abstract]) OR Cancers[Title/Abstract]) OR Malignancy[Title/Abstract]) OR Malignancies[Title/Abstract]) OR Malignant Neoplasms[Title/Abstract]) OR Malignant Neoplasm[Title/Abstract]) OR Neoplasm, Malignant[Title/Abstract]) OR Neoplasms, Malignant[Title/Abstract]) OR Benign Neoplasms[Title/Abstract]) OR Neoplasms, Benign[Title/Abstract]) OR Benign Neoplasm[Title/Abstract]) OR Neoplasm, Benign[Title/Abstract])AND(exosomes[Title/Abstract]) OR exosome[Title/Abstract]). We performed an additional manual search to avoid missing correlatively original literature by using the reference lists of relevant studies.

### 2.2. Selection of Studies

Studies that met these criteria were included: (1) include all types of solid tumor patients; (2) all solid tumor patients were diagnosed by clinical histopathology; (3) included healthy individuals as a control group; (4) provided the expression levels of exosomal lncRNA; (5) provided the method to testify to the existence of related exosomes; (6) evaluated a liquid sample type; (7) included data about the diagnostic significance of exosomes in all types of cancer patients; and (8) included sample size, control group size, and sensitivity and specificity. Studies with the following characteristics were excluded: (1) duplicate literature; (2) insufficient data; (3) meta-analyses, letters, animal experiments, and reviews; (4) noncancer research; (5) articles that did not verify the presence of exosomes; articles that did not have diagnostic significance data related to the exosomal lncRNA; and (7) studies with a control group that did not meet the requirements.

This study was independently implemented through 2 investigators. In the case of discordance, there was discussion with a third researcher to reach a consensus. The inclusion and exclusion of two investigators was established in Table S2. The *k*-agreement was calculated between two investigators when deciding to include exclude articles. The *k*-agreement was 0.942 (*p* < 0.005), which indicated the great inter-rater agreement between the two independent investigators and the included studies meet the selective standards.

### 2.3. Data Extraction

We collected the true-negative, false-negative, true-positive, and false-positive values from the selected literature. For literature that did not provide these data, we calculated these based on the sensitivity and specificity and the number of samples. Moreover, we collected the author name, publication year, country, and ethnicity of the study population, cancer type, lncRNA, sample type, number of the case-patients, and number of control patients, as well as the true positivity, false positivity, true negativity, false negativity, sensitivity, and specificity. Although some articles did not relate clear information, we were able to contact the authors for clarification.

### 2.4. Quality Assessment

According to the Quality Assessment of Diagnosis Accuracy Studies (QUADAS-2) criteria (http://prisma-statement.org), we evaluated the quality of the included studies using RevMan 5.3 software [31]. The QUADAS-2 included 14 questions about the risk of bias of the included article. Answers included yes, not clear, and no, which corresponded to scores of −1, 0, and 1, respectively. We eliminated low-quality studies from our analysis. The quality of the literature was independently assessed by 2 investigators.

### 2.5. Statistical Analysis

Statistical analysis was performed by Stata 14.0 software (Stata, College Station, TX). We applied the bivariate random-effects regression model to combine the effect values of all included studies. The analysis included sensitivity, specificity, diagnostic likelihood ratio negative (DLR-), diagnostic likelihood ratio positive (DLR+) with corresponding 95% confidence intervals (CI) [32].The Higgin's *I*^2^ and Cochran's *Q* tests were also included in the analysis [33]. Moreover, we used the kappa statistic to analyse the concordance between diagnosis on exosomal lncRNA and clinical histopathology. Furthermore, we plotted the bivariate boxplot to roughly assess the heterogeneity of the study. We also plotted the summary receiver operating characteristic curve to calculate the pooled under the curve (AUC) value [34].

To further explore the potential heterogeneity between the included studies, we performed a subgroup analysis by Stata software 14.0. The included studies are divided into 6 subgroups based on tumor type, number of samples, and exosome isolation. We performed pooled SEN, SPE, DLR+, DLR-, diagnostic score, and diagnostic odds ratio analysis. Furthermore, Deek's funnel plot asymmetry test was performed to show the publication bias in the study, with the unequally distributed visual funnel plot or a *p* value less than 0.5 indicating significant publication bias in the study [35].

To explore the more clinical significance of the study, we plotted the Fagan plot showing the relationship between previous probability, likelihood ratio, and posterior probability. We also performed a likelihood ratio scattergram to determine the application value of exosome lncRNA in the clinical diagnosis.

## 3. Results

### 3.1. Characteristics and Quality of the Included Studies

The procedure for selecting the included literature in this study is shown in Figure S1. We obtained 863 potential related articles based on the need for the research and the search criteria of the electronic literature database. Because of repetition, 411 articles were excluded. We then excluded 424 articles by manually screening the title and abstract of the article. We screened the full text and excluded 9 articles that did not focus on the topic (*n* = 3) or had incomplete data (*n* = 6). Finally, we obtained 19 articles. Several studies have been carried out simultaneously in the literature. Yazarlou et al. [36] showed 4 lncRNAs (UCa1-201, UCa1-203, MalaT1, LinC00355) in exosomes. Zhan et al. [37] reported 3 lncRNAs (MALAT1, PCAT-1, SPRY4-IT1) in exosomes. Zhao et al. [38] illustrated 2 lncRNAs (HOTTIP, PTENP1) in exosomes. Wang et al. [39] demonstrated 2 different lncRNAs (SAP30L-AS1, SChLAP1) in exosomes, and Zhang et al. [40] found 3 lncRNAs (PCAT-1, UBC1, SNHG16). Moreover, Qier et al. [41] reported the lncRNA LINC00152 in exosomes, Wang et al. [42] showed the lncRNA HOTAIR in exosomes, Liu et al. [43] showed the lncRNA CRNDE-h in exosomes, Ge et al. [44] illustrated the lncRNA ENST00000588480.1 in exosomes, Zhang et al. [45] reported the lncRNA MALAT-1 in exosomes, Xue et al. [46] reported the lncRNA UCA-1 in exosomes, Dong et al. [47] reported the lncRNA SNHG14 in exosomes, Kang et al. [48] reported the lncRNA PART1 in exosomes, Sun et al. [49] reported the lncRNA LINC00161 in exosomes, Wang et al. [50] reported the lncRNA H19 in exosomes, Xu et al. [51] reported the lncRNA ENSG00000258332.1 in exosomes, Zhang et al. [52] reported the lncRNA RP11-838N2.4 in exosomes, Zhao et al. [38] reported the lncRNA HOTTIP in exosomes, Zheng et al. [53] reported the lncRNA PTENP1 in exosomes, and Teng et al. [54] reported the lncRNA SOX2-OT in exosomes. Thus, 19 articles were selected that included 28 studies with a total of 2084 cancer patients and 1933 controls.

The basic information of these 28 studies is shown in Table 1. These studies were performed between 2014 and 2019. Four studies were conducted in Iran, and 25 studies were conducted in China. The sample size of the studies ranges from 60 to 320. The sample types were plasma in 5 studies, urine in 10 studies, and serum in 14 studies. In addition, there were 10 types of cancer, including bladder cancer (13 studies), gastric cancer, laryngeal squamous cell carcinoma, colorectal cancer, cholangiocarcinoma, breast cancer, esophageal squamous cell carcinoma, hepatocellular carcinoma, nonsmall cell lung cancer, and prostate cancer. We also collected the information of exosomes that were included in the studies in Table 2. This included the isolation method, exosome content, content number, exosomes diameter range (nm), median diameter (nm), evaluation (TEM and NET), and exosome proteins.

### 3.2. Quality Assessments

**Figure 1:**
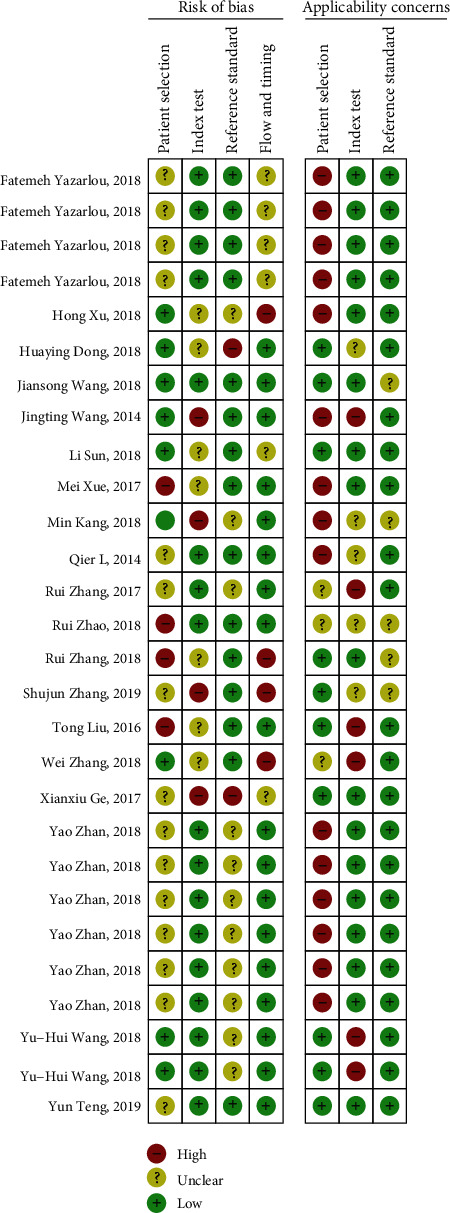
Overall quality assessment of the included articles using the QUADAS-2 tool.

As shown in Figure 1, the outcomes of the QUADAS-2 study quality assessment indicated that the quality of the included studies was convincing. The QUADAS score is shown in Table 1. The titles of all included documents are provided in Table 3. We assumed that a score greater than 4 indicated a high-quality study and a score lower than 4 indicated a study with low quality. We included 8 poor quality studies and 20 better quality studies.

### 3.3. Heterogeneity and Concordance

**Figure 2:**
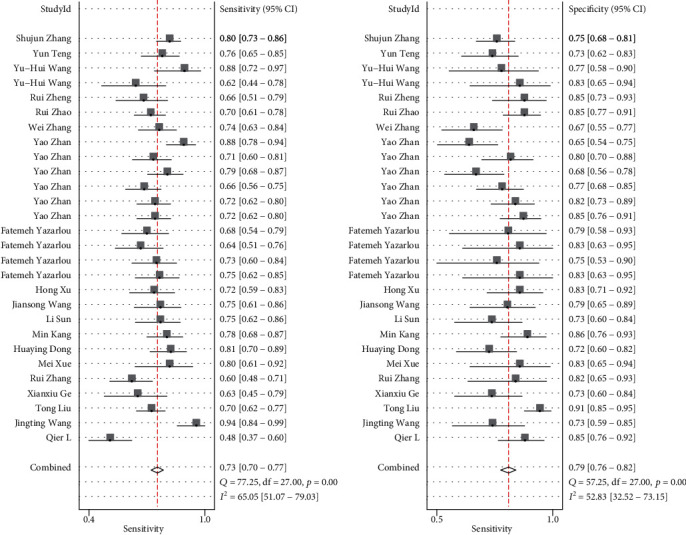
Forest plot of sensitivity and specificity for the diagnosis of lncRNA of liquid exosomes in tumor among 28 studies.

We assessed heterogeneity by using the *p* value of the Moss model from the threshold effect and Spearman's correlation. The Cochran *Q* and *I*^2^ tests were used to evaluate heterogeneity between the included studies. In these studies, the Cochran *Q* was 40.775 (*p* < 0.05), *I*^2^ tests were 95 (95% CI, 91–99). The Cochran *Q* and *I*^2^ tests of sensitivity and specificity (Figure 2) were 77.25 (*p* < 0.05), 65.05 (95% CI, 51.07–79.03), 57.25 (*p* < 0.05), 52.83 (95% CI, 32.62–73.15). These outcomes indicated that significant heterogeneity exists in the included studies. Furthermore, we estimated the heterogeneity through graphic methods by bivariate boxplot, as shown in Figure S2. Most of the research fell in the middle, but 4 studies were outside, which also indicated that there may be heterogeneity between the included studies. Furthermore, we assessed the concordance between diagnosis on exosomal lncRNA and clinical histopathology by using the kappa statistic. The kappa statistic of all studies was 0.513, which shows that exosomal lncRNA is moderately accurate to diagnose solid tumors. Moreover, to further explore the concordance between diagnosis on exosomal lncRNA and clinical histopathology, we analyzed the kappa statistic in different types of solid tumors and different sample types. The concordance of blood samples (kappa statistic, 0.523) was better than that of urine samples (kappa statistic, 0.494). The concordance of digestive system tumors (kappa statistic, 0.542) was better than that of urinary system tumors (kappa statistic, 0.511).

### 3.4. Diagnostic Performance

**Figure 3:**
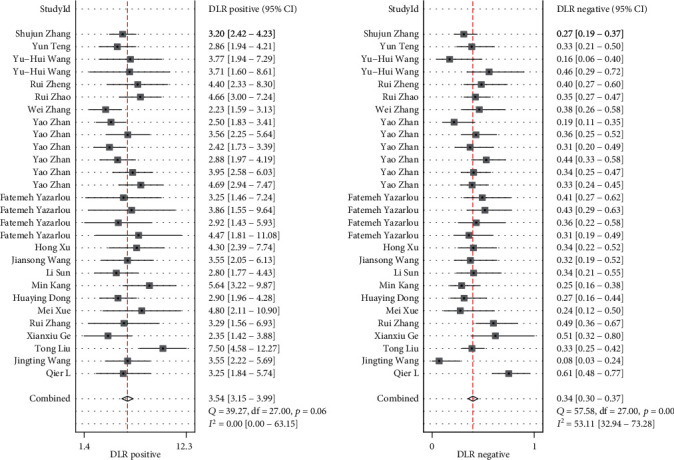
Forest plot of DLR+ and DLR- (DLR: degrees of freedom) for diagnosis of lncRNA of liquid exosomes in tumor among 28 studies.

**Figure 4:**
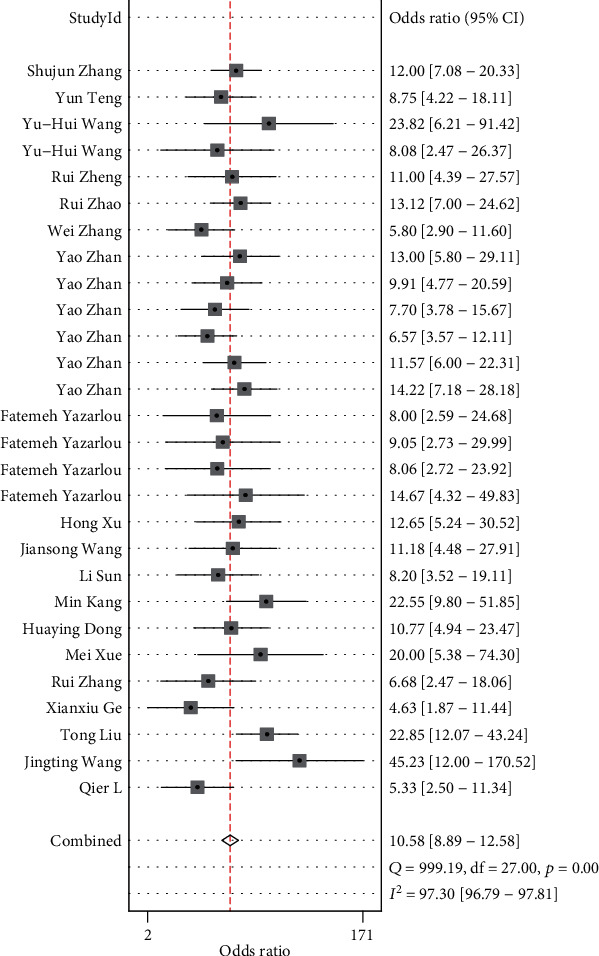
Forest plot of DOR (DOR: diagnostic OR) for diagnosis of lncRNA of liquid exosomes in tumor among 28 studies.

**Figure 5:**
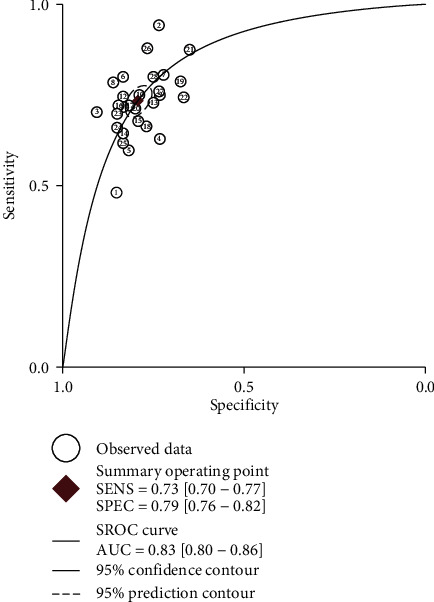
The SROC of the liquid exosomes lncRNA for the diagnosis of cancer. (AUC: area under the curve; SROC: summary receiver operator characteristic).

Sensitivity and specificity are shown in Figure 2 as a forest plot. The combined value of sensitivity in the 29 studies was 0.74 (95% CI, 0.7–0.78), and the combined value of specificity was 0.81 (95% CI, 0.78–0.83). DLR+ and DLR- are shown in Figure 3 as a forest plot. The combined value of DLR+ in the 29 studies was 3.86 (95% CI, 3.31–4.51), and the combined value of DLR-0.32 (95% CI, 0.27–0.37). The pooled odds ratio of the 29 studies was 12.21 (95% CI, 9.31–16), as shown in Figure 4. Furthermore, Figure 5 shows that the AUC was 0.85 (95% CI, 0.81–0.88). These results demonstrated the high diagnostic efficacy of liquid exosomes in cancer patients.

### 3.5. Subgroup Analysis

As shown in Table 4, Figure S3, and Figure S4, we found a difference between digestive system tumors and urinary system tumors in terms of sensitivity (0.73 [95% CI, 0.63–0.81] vs. 0.74 [95% CI, 0.70–0.82]), specificity (0.83 [95% CI, 0.78–0.87] vs. 0.79 [95% CI, 0.75–0.82]), DLR+ (4.21 [95% CI, 3.28–5.41] vs. 3.4 [95% CI, 3.0–4.0]), DLR- (0.33 [95% CI, 0.24–0.45] vs. 0.33 [95% CI, 0.29–0.38]), DOR (12.87 [95% CI, 8.12–20.41] vs. 10 [95% CI, 8–13], and AUC (0.86 [95% CI, 0.82–0.89] vs. 0.83 [95% CI, 0.80–0.86]). In summary, our data illustrated that the diagnostic power is higher in digestive system tumors, the bivariate boxplot (Figure S3E) showed that 6 studies fell in the middle, but 2 studies were outside. The bivariate boxplot (Figure S4E) showed that 14 studies fell in the middle, but 1 study was outside, which also indicated that the heterogeneity between the digestive system tumor group was more significant than that of the urinary system tumor group. We also found a difference between the different sample types in Figure S5, Figure S6, and Table 4. Blood samples had higher sensitivity (0.74), specificity (0.80), and AUC (0.84), indicating that blood samples were more reliable than urine samples. The bivariate boxplot (Figure S5E) showed that 15 studies fell in the middle, but 3 studies were outside. The bivariate boxplot (Figure S6E) showed that 8 studies fell in the middle, but 2 studies were outside. This also indicated that the heterogeneity between the urine groups was more significant than the blood group. Moreover, we found that the group with larger sample sizes (>70) (Figure S7G) and the subgroup with fewer samples (<70) (Figure S8G) had similar capabilities with the same AUC of 0.83 (95% CI, 0.79–0.86). We also performed the subgroup analysis based on other basic information such as age, sex, ethnicity, and country. We found that there were no significant differences between the groups, so they were not discussed.

### 3.6. Publication Bias

**Figure 6:**
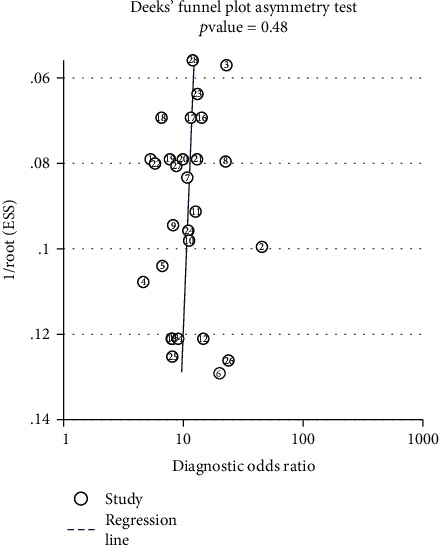
Deek's funnel plot asymmetry test for assessing publication bias.

As shown in Figure 6, the *p* value of the Deek's funnel plot asymmetry test was 0.48, which is greater than 0.05, indicating that these studies did not have publication bias.

### 3.7. Clinical Significance of the Study

**Figure 7:**
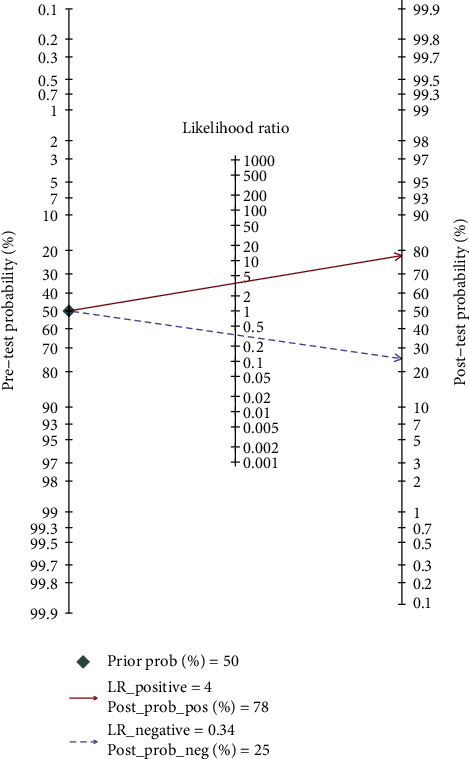
Fagan plot to show the clinical significance in the cancer diagnosis of lncRNA of liquid exosomes.

**Figure 8:**
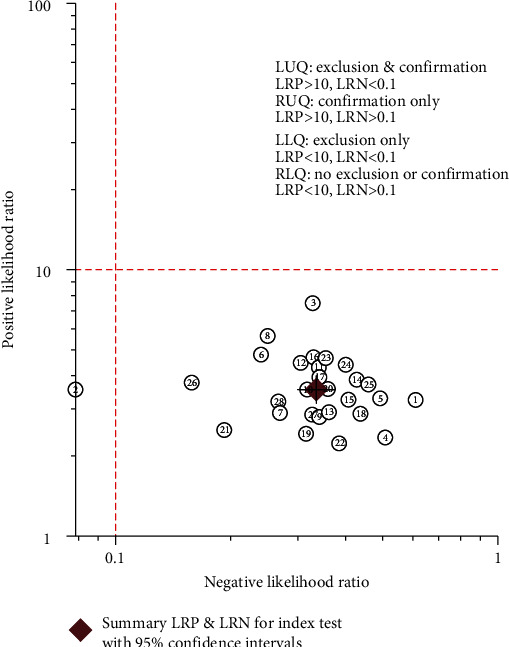
The dot plot of negative likelihood ratio to show the clinical significance in the cancer diagnosis of lncRNA of liquid exosomes.

To explore the clinical significance of this study, the Fagan plot (Figure 7) was performed to show the prior probability of 50%, likelihood ratio of 4, and the post probability of 73%. Furthermore, we plotted the likelihood ratio dot plot (Figure 8). The upper left limit LRP was greater than 10, and the LRN was less than 0.1, indicating that the diagnosis could be diagnosed and excluded. An upper right limit LRP greater than 10 and the LRN greater than 0.1 suggested that the diagnosis could be confirmed. A lower left limit LRP less than 10 and LRN less than 0.1 indicated that the diagnosis could be excluded. A lower right limit LRP less than 10 and LRN greater than 0.1 indicated that neither diagnosis nor diagnosis could be ruled out. In conclusion, it is undeniable that lncRNAs in exosomes could be promising biomarkers in the diagnosis of tumors.

## 4. Discussion

However, there was controversy with respect of the clinical value regarding whether lncRNA could be useful molecules in several cancers. These findings demonstrate that lncRNAs in exosomes can be a compelling indicator of prognosis in human solid tumors, but further confirmation by dependable analyses is required. Therefore, a meta-analysis was performed to account for whether it played a critical role in diagnosis for cancer patients.

This systematic literature review and meta-analysis included 19 articles with a total of 4017 patients with 10 types of malignant tumors. The combined value of sensitivity in 29 studies was 0.74 (95% CI, 0.7–0.78), and the combined value of specificity was 0.81 (95% CI, 0.78–0.83). The combined value of DLR+ in 29 studies was 3.86 (95% CI, 3.31–4.51), and the combined value of DLR- was 0.32 (95% CI, 0.27–0.37). The pooled odds ratio of the 29 studies was 12.21 (95% CI, 9.31–16). The AUC was 0.85 (95% CI, 0.81–0.88). The kappa statistic of all studies was 0.513, which shows that exosomal lncRNA has moderate concordance in diagnosing solid tumors. These results demonstrated the high diagnostic efficacy of liquid exosomes in cancer patients. In our report, the lncRNAs in exosomes were defined by an authoritative method, thus our results are credible. The outcome indicates that there may be heterogeneity between the included studies. And, the results of sensitivity, specificity, and DLR show they can be highly diagnostic efficacy biomarkers. In addition, we conducted subgroup analyses including sex, ethnicity, year, sample type, sample number, and cancer type, which showed no statistical significance in terms of sex, ethnicity, and year. The diagnostic power of digestive system tumors was found to be higher than that of urinary system tumors (sensitivity, 0.73 [95% CI, 0.63–0.81] vs. 0.74 [95% CI, 0.70–0.82]; specificity, 0.83 [95% CI, 0.78–0.87] vs. 0.79 [95% CI, 0.75–0.82]; DLR+, 4.21 [95% CI, 3.28–5.41] vs. 3.4 [95% CI, 3.0–4.0]; DLR-, 0.33 [95% CI, 0.24–0.45] vs. 0.33 [95% CI, 0.29–0.38]; DOR, 12.87 [95% CI, 8.12–20.41] vs. 10 [95% CI, 8–13]; and AUC, 0.86 [95% CI, 0.82–0.89] vs. 0.83 [95% CI, 0.80–0.86]). Thus, the potential biological mechanisms of exosomal lncRNA in the digestive system should be studied further in future studies. The bivariate boxplot indicated that the heterogeneity of the digestive system tumor group was more significant than that of the urinary system group. Moreover, our outcome also suggested that the heterogeneity between the blood group was more significant than that of the urine group, suggesting that we could adopt a blood-exosomes isolation strategy in our further research to collect better data.

With the development of biomarkers in cancer therapy, more specific and high-efficiency molecules have been used clinically, such as circulating tumor DNA (ctDNA), circulating tumor cells, microRNA, lncRNA, and circRNA. For instance, Lee et al. illustrated that patients with detectable ctDNA, a promising prognostic biomarker, showed a trend toward higher risk for disease recurrence than those without detectable ctDNA [55]. Circulating tumor cells can enable early cancer detection, tumor dynamics assessment, minimal residual disease detection, and therapy monitoring [56]. Our study indicated that mir-24-3p, a type of miRNA, can promote cell migration and invasion by targeting TEL2 in nasopharyngeal carcinoma as a biomarker [57]. SNHG1, a novel lncRNA of the SNHG family, indicated that SNHG1 could be a compelling prognosis indicator in human solid tumors [29]. Compared with traditional biomarkers such as AFP [58], CEA [59], and CA199 [60], these next-generation molecules represent high potential in diagnosis value. Nonetheless, the novel biomarkers we mentioned previously still need to be further verified in the future. Therefore, as clinical researchers, it is our duty to explore novel biomarkers and cells that help doctors determine the clinical strategy for cancer patients.

**Figure 9:**
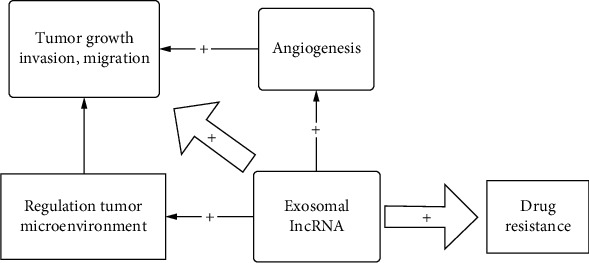
The potential biological mechanisms of exosomal lncRNA. Exosomes can directly promote tumor growth, invasion, migration, and drug resistance. And indirectly promote tumor growth, invasion, migration by promoting angiogenesis, and regulate microenvironment.

Additionally, our team was also the first to summarize the decade of exosomal long RNA species, suggesting the significant role of exosomal lncRNA in cancer diagnosis and clinical therapy. Accumulating reports have illustrated that exosomal lncRNA is related to many processes of cancer deterioration, such as tumor cell-cell communication, drug resistance, invasion, migration, immune response, and angiogenesis [61]. Wu et al. revealed that the addition of epithelial ovarian cancer-derived exomes in the coculture system, by transferring lncRNAs, restored the migration of endothelial cells which had been inhibited by TAM-derived exosomes through targeting the miR-146b-5p/TRAF6/NF-*κ*B/MMP2 pathway, which suggests that exosomal lncRNAs play a powerful role in the regulation of the tumor microenvironment [62]. Moreover, some studies have reported that exosomal lncRNA can regulate the tumor microenvironment to influence the invasion, growth, metastasis, and prognosis of tumors [63, 64]. Other research has also found that exosomal lncRNA can induce angiogenesis in cancer cells and affect tumor prognosis, growth, and invasion [65, 66]. Pan et al. also found that exosomal lncRNA was additionally related to lymphatic metastasis in gastric cancer patients [67], which showed that tumors can indirectly enhance cellular migration and invasion abilities through exosomal lncRNA. Moreover, some studies have revealed that exosomal lncRNA plays a key role in the acquired drug resistance in some cancers [68, 69]. Takahashi et al. found that LINC-ROR, derived from tumor cells, was enriched in the extracellular vesicles, which can lead to an increased level of chemoresistance in hepatocellular cancer cells. This suggests that hepatocellular cancer cells may use exosomal lncRNA to improve chemoresistance [70]. The potential biological mechanisms of exosomal lncRNA are summarized in Figure 9. Moreover, one of the most significant previous findings in exosomes for our report is that exosomes demonstrated a cargo biological function to deliver a number of molecules, such as drug, ncRNAs, and mRNAs [61]. Therefore, this special biological feature has led to many questions and a need for further investigation. Further work needs to be done to establish whether exosomes are involved in malignant tumor invasion as well as migration. Moreover, because of the specificity [71], stability [72], and accessibility [73], we can not only use exosomes to diagnose solid tumors but we can also apply them to gene therapy.

## 5. Conclusions

As a type of special molecule, lncRNA in exosomes could be a promising bioindicator for the diagnosis and prognosis of cancers. Our study provides convincing evidence through meta-analysis. However, further works are required in the future. Finally, we hope our results encourage more researchers to examine the prognostic and diagnostic role of lncRNA in exosomes as well as explore the underlying biomechanisms in different cancers.

## Figures and Tables

**Table 1:** Characteristics of the included studies.

Author	Year	Country	Ethnic	Cancer type	lncRNA	Sample	Case	Control	TP	FP	FN	TN	Sensitivity (%)	Specificity (%)	QUADAS score
Qier L	2014	China	Asian	GC	LINC00152	Plasma	79	81	38	12	41	69	0.481	0.852	4
Jiangting Wang	2014	China	Asian	LSCC	HOTAIR	Serum	52	49	49	13	3	36	0.942	0.735	4
Tong Liu	2016	China	Asian	CC	CRNDE-h	Serum	148	160	104	15	44	145	0.703	0.904	4
Xianxiu Ge	2017	China	Asian	CGC	ENST00000588480.1	Serum	35	56	22	15	13	41	0.629	0.732	3
Rui Zhang	2017	China	Asian	NSCLC	MALAT-1	Serum	77	33	46	6	31	27	0.601	0.809	3
Mei Xue	2017	China	Asian	BD	UCA1	Serum	30	30	24	5	6	25	0.800	0.833	4
Huaying Dong	2018	China	Asian	BC	SNHG14	Serum	72	72	58	20	14	52	0.800	0.725	4
Min Kang	2018	China	Asian	ESCC	PART1	Serum	79	79	62	11	17	68	0.786	0.865	2
Li Sun	2018	China	Asian	HCC	LINC00161	Serum	56	56	42	15	14	41	0.750	0.732	5
Jiansong Wang	2018	China	Asian	BC	H19	Serum	52	52	39	11	13	41	0.741	0.781	6
Hong Xu	2018	China	Asian	HCC	ENSG00000258332.1	Serum	60	60	43	10	17	50	0.716	0.834	3
Fatemeh Yazarlou	2018	Iran	Asian	BD	UCa1-201	Urine	59	24	44	4	15	20	0.754	0.812	4
Fatemeh Yazarlou	2018	Iran	Asian	BD	UCa1-203	Urine	59	24	43	6	16	18	0.724	0.750	4
Fatemeh Yazarlou	2018	Iran	Asian	BD	MalaT1	Urine	59	24	38	4	21	20	0.638	0.833	4
Fatemeh Yazarlou	2018	Iran	Asian	BD	LinC00355	Urine	59	24	40	5	19	19	0.680	0.792	4
Yao Zhan	2018	China	Asian	BD	MALAT1	Urine	104	104	75	16	29	88	0.721	0.846	4
Yao Zhan	2018	China	Asian	BD	PCAT-1	Urine	104	104	75	19	29	85	0.721	0.817	4
Yao Zhan	2018	China	Asian	BD	SPRY4-IT1	Urine	104	104	69	24	35	80	0.663	0.769	4
Yao Zhan	2018	China	Asian	BD	MALAT1	Urine	80	80	63	26	17	54	0.787	0.675	4
Yao Zhan	2018	China	Asian	BD	PCAT-1	Urine	80	80	57	16	23	64	0.712	0.800	4
Yao Zhan	2018	China	Asian	BD	SPRY4-IT1	Urine	80	80	70	28	10	52	0.875	0.650	4
Wei Zhang	2018	China	Asian	NSCLC	RP11-838N2.4	Serum	78	78	58	26	20	52	0.745	0.673	3
Rui Zhao	2018	China	Asian	GC	HOTTIP	Serum	126	120	88	18	38	102	0.698	0.85	3
Rui Zheng	2018	China	Asian	BD	PTENP1	Plasma	50	60	33	9	17	51	0.654	0.842	3
Yu-Hui Wang	2018	China	Asian	PSA	SAP30L-AS1	Plasma	34	30	21	5	13	25	0.611	0.821	5
Yu-Hui Wang	2018	China	Asian	PSA	SChLAP1	Plasma	33	30	29	7	4	23	0.879	0.767	5
Yun Teng	2019	China	Asian	LSCC	SOX2-OT	Plasma	75	79	57	21	18	58	0.760	0.732	6
Shujun Zhang	2019	China	Asian	BD	PCAT-1, UBC1, SNHG16	Serum	160	160	128	40	32	120	0.800	0.750	2

GC: gastric cancer; LSCC: laryngeal squamous cell carcinoma; CC: colorectal cancer; CGC: cholangiocarcinoma; NSCLC: nonsmall cell lung cancer; BD: bladder tumor; BC: breast cancer; ESCC: esophageal squamous cell carcinoma; HCC: hepatocellular carcinoma; PSA: prostate cancer; TP: true positivity; FP: false positivity; TN: true negativity; FN: false negativity.

**Table 2:** The information for the exosome of the included studies.

Author	Year	Isolation method	Exosome content	Content number	Exosomes diameter range(nm)	Median diameter(nm)	Evaluation		Exosome proteins	TEM	NTA
							TEM (nM)	NTA (nM)			
Qier L	2014	Total exosome isolation kit	lncRNA	1	30-110	None	Yes	None	None	None	None
Jingting Wang	2014	ExoQuick precipitation kit	lncRNA	1	40-100	None	Yes	None	CD63	/	/
Tong Liu	2016	ExoQuick precipitation kit	lncRNA	1	40-100	100	Yes	None	CD63, Hsp70	None	None
Xianxiu Ge	2017	None	lncRNA	1	30-110	None	Yes	Yes	CD63, CD81	30-150	72.2
Rui Zhang	2017	ExoQuick TC	lncRNA	1	30-120	None	Yes	Yes	CD9, CD63	/	/
Mei Xue	2017	ExoQuick precipitation kit	lncRNA	1	50-200	None	Yes	Yes	CD63, TSG101, Hsp70,Hsp90	50-200	None
Huaying Dong	2018	ExoQuick precipitation kit	lncRNA	1	40-120	100	Yes	Yes	CD63, CD81	None	None
Min Kang	2018	ExoQuick precipitation kit	lncRNA	1	30-100	57.89	Yes	Yes	CD63, CD82	20-200	None
Li Sun	2018	Total exosome isolation kit	lncRNA	1	30-150	None	Yes	None	CD63	None	None
Jiansong Wang	2018	ExoQuick precipitation kit	lncRNA	1	30-150	67.52	Yes	None	TSG101, Hsp70	None	None
Hong Xu	2018	Total exosome isolation kit	lncRNA	1	30-100	None	None	None	None	None	None
Fatemeh Yazarlou	2018	Norgen's urine exosome RNA isolation kit	lncRNA	4	240	None	None	None	CD63	None	None
Yao Zhan	2018	Urine exosome RNA isolation kit	lncRNA	3	60-150	None	Yes	Yes	CD63, CD81	60-150	20-200
Wei Zhang	2018	ExoQuick precipitation kit	lncRNA	1	30-150	57.89	Yes	None	CD9	20-200	None
Rui Zhao	2018	None	lncRNA	1	50-150	None	Yes	Yes	None	/	/
Rui Zheng	2018	ExoQuick exosome precipitation solution	lncRNA	1	50-120	None	Yes	None	CD63, TSG101	None	None
Yu-Hui Wang	2018	None	lncRNA	2	30-120	None	Yes	None	CD63, TSG101	None	None
Yun Teng	2019	None	lncRNA	1	30-100	None	Yes	None	CD9, CD63	None	None
Shujun Zhang	2019	ExoQuick™ solution	lncRNA	3	100	None	Yes	Yes	CD9, CD63	None	None

**Table 3:** Bibliographic information of the included articles.

Number	Title
1	Exosomal long noncoding RNA HOTTIP as potential novel diagnostic and prognostic biomarker test for gastric cancer
2	Exosome-mediated transfer of lncRNA RP11-838N2.4 promotes erlotinib resistance in nonsmall cell lung cancer
3	Evaluation of serum exosomal lncRNA-based biomarker panel for diagnosis and recurrence prediction of bladder cancer
4	Expression signatures of exosomal long non-coding RNAs in urine serve as novel noninvasive biomarkers for diagnosis and recurrence prediction of bladder cancer
5	Urinary exosomal expression of long noncoding RNAs as diagnostic marker in bladder cancer
6	Hypoxic exosomes facilitate bladder tumor growth and development through transferring long noncoding RNA-UCA1
7	Serum exosomal long noncoding RNAs ENSG00000258332.1 and LINC00635 for the diagnosis and prognosis of hepatocellular carcinoma
8	Combined detection of serum exosomal miR-21 and HOTAIR as diagnostic and prognostic biomarkers for laryngeal squamous cell carcinoma
9	Determination of serum exosomal H19 as a noninvasive biomarker for bladder cancer diagnosis and prognosis
10	Serum and exosome long non coding RNAs as potential biomarkers for hepatocellular carcinoma
11	Exosomal long noncoding RNA CRNDE-h as a novel serum-based biomarker for diagnosis and prognosis of colorectal cancer
12	Plasma long noncoding RNA protected by exosomes as a potential stable biomarker for gastric cancer
13	Exosome-mediated transfer of lncRNA PART1 induces gefitinib resistance in esophageal squamous cell carcinoma via functioning as a competing endogenous RNA
14	The diagnostic/prognostic potential and molecular functions of long noncoding RNAs in the exosomes derived from the bile of human cholangiocarcinoma
15	Exosome-mediated transfer of lncRNA-SNHG14 promotes trastuzumab chemoresistance in breast cancer
16	Exosome–transmitted long noncoding RNA PTENP1 suppresses bladder cancer progression
17	Serum long noncoding RNA MALAT-1 protected by exosomes is upregulated and promotes cell proliferation and migration in nonsmall cell lung cancer
18	Tumor-derived exosomal long noncoding RNAs as promising diagnostic biomarkers for prostate cancer
19	Identification of an exosomal long noncoding RNA SOX2-OT in plasma as a promising biomarker for lung squamous cell carcinoma

**Table 4:** Summary results of subgroup for liquid exosomes in the diagnosis of cancer.

Subgroup	Number of studies	Sensitivity (95% CI)	Specificity (95% CI)	DLR+ (95% CI)	DLR- (95% CI)	DOR (95% CI)	AUC (95% CI)
Type of cancer							
Digestive system tumor	8	0.73 (0.63-0.81)	0.83 (0.78-0.87)	4.21 (3.28-5.41)	0.33 (0.24-0.45)	12.87 (8.12-20.41)	0.86 (0.82-0.89)
Urinary system tumor	15	0.74 (0.70-0.78)	0.79 (0.75-0.82)	3.4 (3.0-4.0)	0.33 (0.29-0.38)	10 (8-13)	0.83 (0.80-0.86)
Type of specimen							
Blood	18	0.74 (0.69-0.78)	0.80 (0.76-0.83)	3.7 (3.1-4.3)	0.33 (0.28-0.39)	11 (9-14)	0.84 (0.81-0.87)
Urine	10	0.73 (0.69-0.78)	0.78 (0.73-0.82)	3.3 (2.7-4.0)	0.34 (0.30-0.40)	10 (7-12)	0.82 (0.78-0.85)
Sample size							
>70	15	0.73 (0.69-0.77)	0.79 (0.75-0.83)	3.5 (3.0-4.2)	0.34 (0.29-0.39)	10 (8-13)	0.83 (0.79-0.86)
<70	13	0.74 (0.68-0.79)	0.79 (0.75-0.83)	3.5 (3.0-4.2)	0.33 (0.27-0.40)	11 (8-15)	0.83 (0.79-0.86)

DLR: degrees of freedom; DOR: diagnostic OR; AUC: area under the curve.

## Data Availability

The data supporting the research are included in the article.

## Abbreviation

lncRNAs: Long noncoding RNAs
DM: Distant metastasis
LNM: Lymph node metastasis
TP: True positivity
FP: False positivity
TN: True negativity
FN: False negativity
DLR-: Diagnostic likelihood ratio negative
DLR+: Diagnostic likelihood ratio positive
ctDNA: Circulating tumor DNA
CTC: Circulating tumor cell
miRNA: microRNA
GC: Gastric cancer
LSCC: Laryngeal squamous cell carcinoma
CRC: Colorectal cancer
BC: Breast cancer
ESCC: Esophageal squamous cell carcinoma
HCC: Hepatocellular carcinoma
NSCLC: Nonsmall cell lung cancer
PSA: Prostate cancer.

## Supplementary Materials

Supplementary Materials Figure S1: the study selection procedure for meta-analysis was conducted to the PRISMA statement. Supplementary 2. Table S1: PRISMA Checklist. Supplementary 3. Table S2: the inclusion and exclusion of two investigators. Supplementary 4. Figure S2: the Bivariate Boxplot of the 28 included study to assess the heterogeneity in the included studies. Supplementary 5. Figure S3: forest plot of sensitivity, specificity, DLR+, DLR-, DOR, and the Bivariate Boxplot an SROC of the liquid exosome lncRNA for the diagnosis of digestive system tumor among 8 studies. (A) Sensitivity, (B) specificity, (C) DLR+, (D) DLR-, (F) Bivariate Boxplot, (G) SROC. Supplementary 6. Figure S4: forest plot of sensitivity, specificity, DLR+, DLR-, DOR, and the Bivariate Boxplot an SROC of the liquid exosomes lncRNA for the diagnosis of urinary system tumor among 15 studies. (A) Sensitivity, (B) specificity, (C) DLR+, (D) DLR-, (F) Bivariate Boxplot, (G) SROC. Supplementary 7. Figure S5: forest plot of sensitivity, specificity, DLR+, DLR-, DOR, and the Bivariate Boxplot an SROC of the liquid exosomes lncRNA for the diagnosis in the group of blood sample type among 18 studies. (A) Sensitivity, (B) specificity, (C) DLR+, (D) DLR-, (F) Bivariate Boxplot, (G) SROC. Supplementary 8. Figure S6: forest plot of sensitivity, specificity, DLR+, DLR-, DOR, and the Bivariate Boxplot an SROC of the liquid exosome lncRNA for the diagnosis in the group of urine sample type among 10 studies. (A) Sensitivity, (B) specificity, (C) DLR+, (D) DLR-, (F) Bivariate Boxplot, (G) SROC. Supplementary 9. Figure S7: forest plot of sensitivity, specificity, DLR+, DLR-, DOR, and the Bivariate Boxplot an SROC of the liquid exosome lncRNA for the diagnosis in the group of sample size > 70 among 15 studies. (A) Sensitivity, (B) specificity, (C) DLR+, (D) DLR-, (F) Bivariate Boxplot, (G) SROC. Supplementary 10. Figure S8: forest plot of sensitivity, specificity, DLR+, DLR-, DOR, and the Bivariate Boxplot an SROC of the liquid exosome lncRNA for the diagnosis in the group of blood sample type among 13 studies. (A) Sensitivity, (B) specificity, (C) DLR+, (D) DLR-, (F) Bivariate Boxplot, (G) SROC.
