# Supplementary figures and images for: The Potential Diagnostic Value of Exosomal Long Noncoding RNAs in Solid Tumors: A Meta-Analysis and Systematic Review
Source: Biomed Res Int. 2020 Aug 15;2020:6786875. doi: 10.1155/2020/6786875 (PMC7448226; doi:10.1155/2020/6786875)

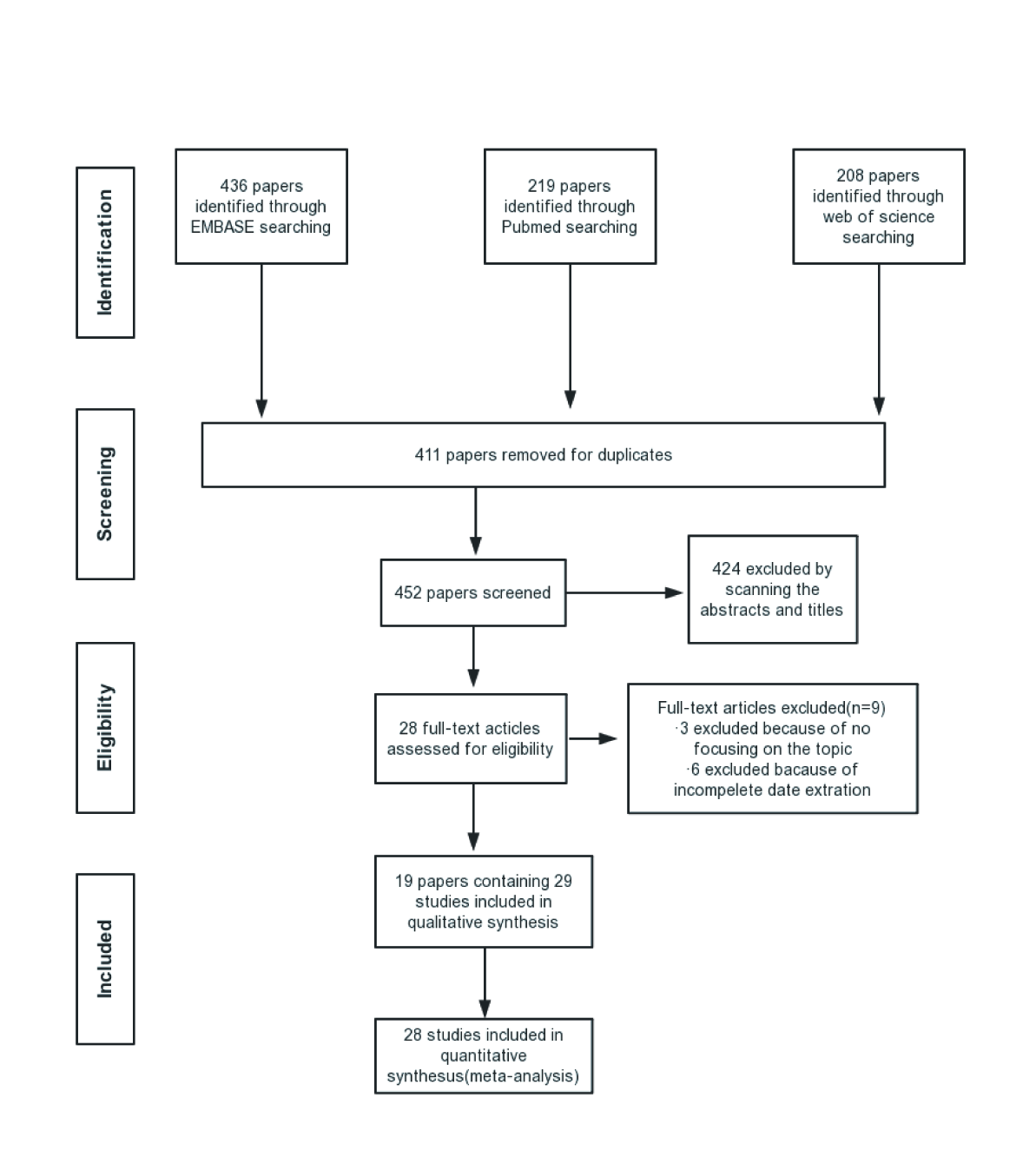

Supplement: Supplementary Materials — Figure S1: the study selection procedure for meta-analysis was conducted to the PRISMA statement. Supplementary 2. Table S1: PRISMA Checklist. Supplementary 3. Table S2: the inclusion and exclusion of two investigators. Supplementary 4. Figure S2: the Bivariate Boxplot of the 28 included study to assess the heterogeneity in the included studies. Supplementary 5. Figure S3: forest plot of sensitivity, specificity, DLR+, DLR-, DOR, and the Bivariate Boxplot an SROC of the liquid exosome lncRNA for the diagnosis of digestive system tumor among 8 studies. (A) Sensitivity, (B) specificity, (C) DLR+, (D) DLR-, (F) Bivariate Boxplot, (G) SROC. Supplementary 6. Figure S4: forest plot of sensitivity, specificity, DLR+, DLR-, DOR, and the Bivariate Boxplot an SROC of the liquid exosomes lncRNA for the diagnosis of urinary system tumor among 15 studies. (A) Sensitivity, (B) specificity, (C) DLR+, (D) DLR-, (F) Bivariate Boxplot, (G) SROC. Supplementary 7. Figure S5: forest plot of sensitivity, specificity, DLR+, DLR-, DOR, and the Bivariate Boxplot an SROC of the liquid exosomes lncRNA for the diagnosis in the group of blood sample type among 18 studies. (A) Sensitivity, (B) specificity, (C) DLR+, (D) DLR-, (F) Bivariate Boxplot, (G) SROC. Supplementary 8. Figure S6: forest plot of sensitivity, specificity, DLR+, DLR-, DOR, and the Bivariate Boxplot an SROC of the liquid exosome lncRNA for the diagnosis in the group of urine sample type among 10 studies. (A) Sensitivity, (B) specificity, (C) DLR+, (D) DLR-, (F) Bivariate Boxplot, (G) SROC. Supplementary 9. Figure S7: forest plot of sensitivity, specificity, DLR+, DLR-, DOR, and the Bivariate Boxplot an SROC of the liquid exosome lncRNA for the diagnosis in the group of sample size > 70 among 15 studies. (A) Sensitivity, (B) specificity, (C) DLR+, (D) DLR-, (F) Bivariate Boxplot, (G) SROC. Supplementary 10. Figure S8: forest plot of sensitivity, specificity, DLR+, DLR-, DOR, and the Bivariate Boxplot an SROC of the liquid exoso [file 6786875.f1.zip › Fig S1.tif]

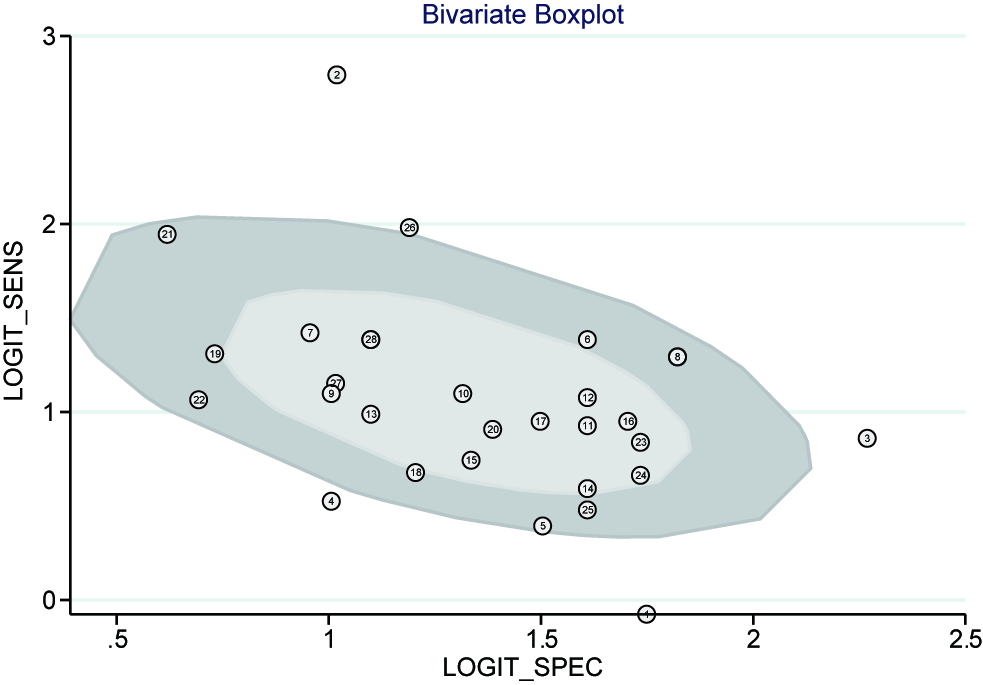

Supplement: Supplementary Materials — Figure S1: the study selection procedure for meta-analysis was conducted to the PRISMA statement. Supplementary 2. Table S1: PRISMA Checklist. Supplementary 3. Table S2: the inclusion and exclusion of two investigators. Supplementary 4. Figure S2: the Bivariate Boxplot of the 28 included study to assess the heterogeneity in the included studies. Supplementary 5. Figure S3: forest plot of sensitivity, specificity, DLR+, DLR-, DOR, and the Bivariate Boxplot an SROC of the liquid exosome lncRNA for the diagnosis of digestive system tumor among 8 studies. (A) Sensitivity, (B) specificity, (C) DLR+, (D) DLR-, (F) Bivariate Boxplot, (G) SROC. Supplementary 6. Figure S4: forest plot of sensitivity, specificity, DLR+, DLR-, DOR, and the Bivariate Boxplot an SROC of the liquid exosomes lncRNA for the diagnosis of urinary system tumor among 15 studies. (A) Sensitivity, (B) specificity, (C) DLR+, (D) DLR-, (F) Bivariate Boxplot, (G) SROC. Supplementary 7. Figure S5: forest plot of sensitivity, specificity, DLR+, DLR-, DOR, and the Bivariate Boxplot an SROC of the liquid exosomes lncRNA for the diagnosis in the group of blood sample type among 18 studies. (A) Sensitivity, (B) specificity, (C) DLR+, (D) DLR-, (F) Bivariate Boxplot, (G) SROC. Supplementary 8. Figure S6: forest plot of sensitivity, specificity, DLR+, DLR-, DOR, and the Bivariate Boxplot an SROC of the liquid exosome lncRNA for the diagnosis in the group of urine sample type among 10 studies. (A) Sensitivity, (B) specificity, (C) DLR+, (D) DLR-, (F) Bivariate Boxplot, (G) SROC. Supplementary 9. Figure S7: forest plot of sensitivity, specificity, DLR+, DLR-, DOR, and the Bivariate Boxplot an SROC of the liquid exosome lncRNA for the diagnosis in the group of sample size > 70 among 15 studies. (A) Sensitivity, (B) specificity, (C) DLR+, (D) DLR-, (F) Bivariate Boxplot, (G) SROC. Supplementary 10. Figure S8: forest plot of sensitivity, specificity, DLR+, DLR-, DOR, and the Bivariate Boxplot an SROC of the liquid exoso [file 6786875.f1.zip › Figure S2.tif]

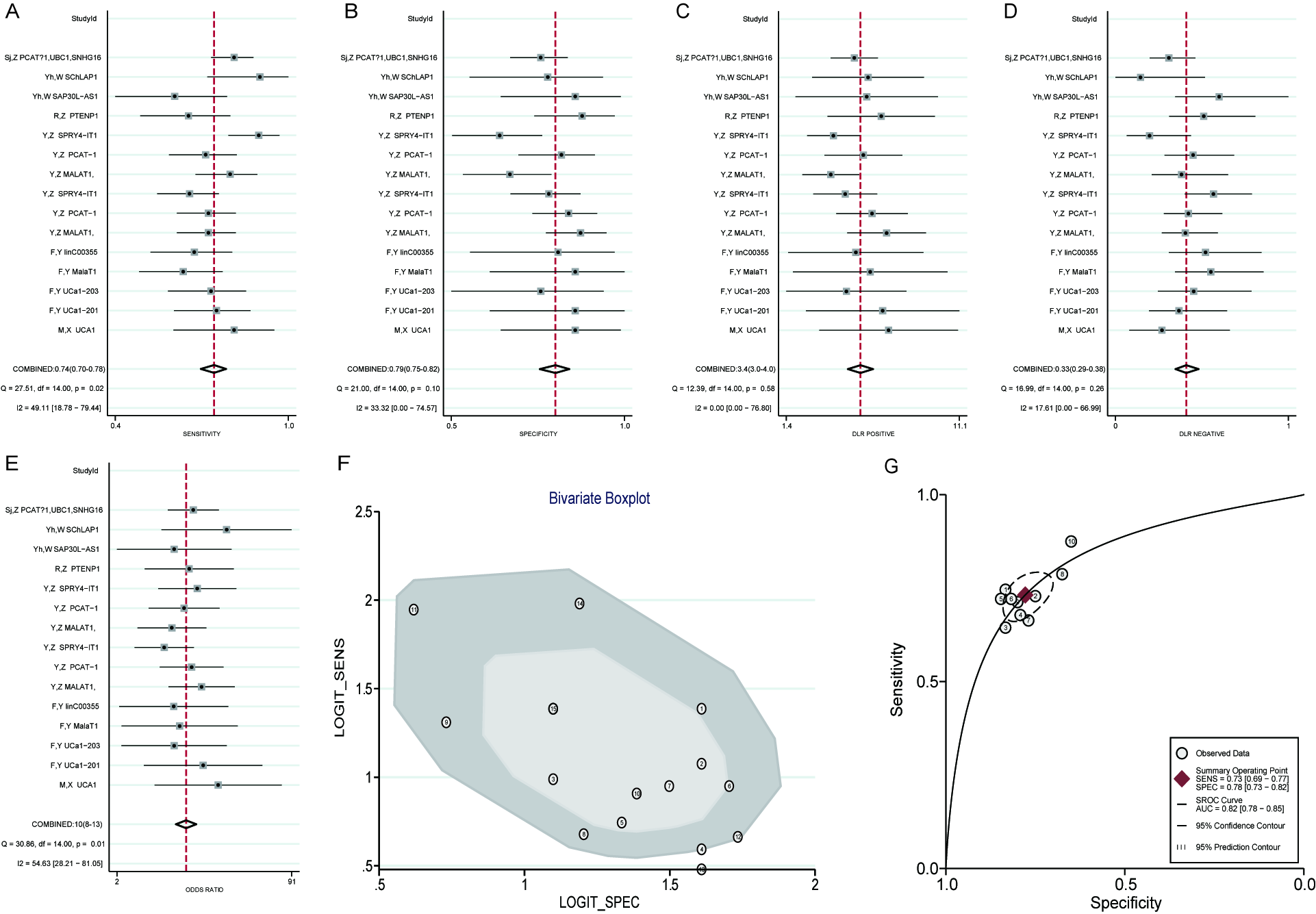

Supplement: Supplementary Materials — Figure S1: the study selection procedure for meta-analysis was conducted to the PRISMA statement. Supplementary 2. Table S1: PRISMA Checklist. Supplementary 3. Table S2: the inclusion and exclusion of two investigators. Supplementary 4. Figure S2: the Bivariate Boxplot of the 28 included study to assess the heterogeneity in the included studies. Supplementary 5. Figure S3: forest plot of sensitivity, specificity, DLR+, DLR-, DOR, and the Bivariate Boxplot an SROC of the liquid exosome lncRNA for the diagnosis of digestive system tumor among 8 studies. (A) Sensitivity, (B) specificity, (C) DLR+, (D) DLR-, (F) Bivariate Boxplot, (G) SROC. Supplementary 6. Figure S4: forest plot of sensitivity, specificity, DLR+, DLR-, DOR, and the Bivariate Boxplot an SROC of the liquid exosomes lncRNA for the diagnosis of urinary system tumor among 15 studies. (A) Sensitivity, (B) specificity, (C) DLR+, (D) DLR-, (F) Bivariate Boxplot, (G) SROC. Supplementary 7. Figure S5: forest plot of sensitivity, specificity, DLR+, DLR-, DOR, and the Bivariate Boxplot an SROC of the liquid exosomes lncRNA for the diagnosis in the group of blood sample type among 18 studies. (A) Sensitivity, (B) specificity, (C) DLR+, (D) DLR-, (F) Bivariate Boxplot, (G) SROC. Supplementary 8. Figure S6: forest plot of sensitivity, specificity, DLR+, DLR-, DOR, and the Bivariate Boxplot an SROC of the liquid exosome lncRNA for the diagnosis in the group of urine sample type among 10 studies. (A) Sensitivity, (B) specificity, (C) DLR+, (D) DLR-, (F) Bivariate Boxplot, (G) SROC. Supplementary 9. Figure S7: forest plot of sensitivity, specificity, DLR+, DLR-, DOR, and the Bivariate Boxplot an SROC of the liquid exosome lncRNA for the diagnosis in the group of sample size > 70 among 15 studies. (A) Sensitivity, (B) specificity, (C) DLR+, (D) DLR-, (F) Bivariate Boxplot, (G) SROC. Supplementary 10. Figure S8: forest plot of sensitivity, specificity, DLR+, DLR-, DOR, and the Bivariate Boxplot an SROC of the liquid exoso [file 6786875.f1.zip › Figure S3.tif]

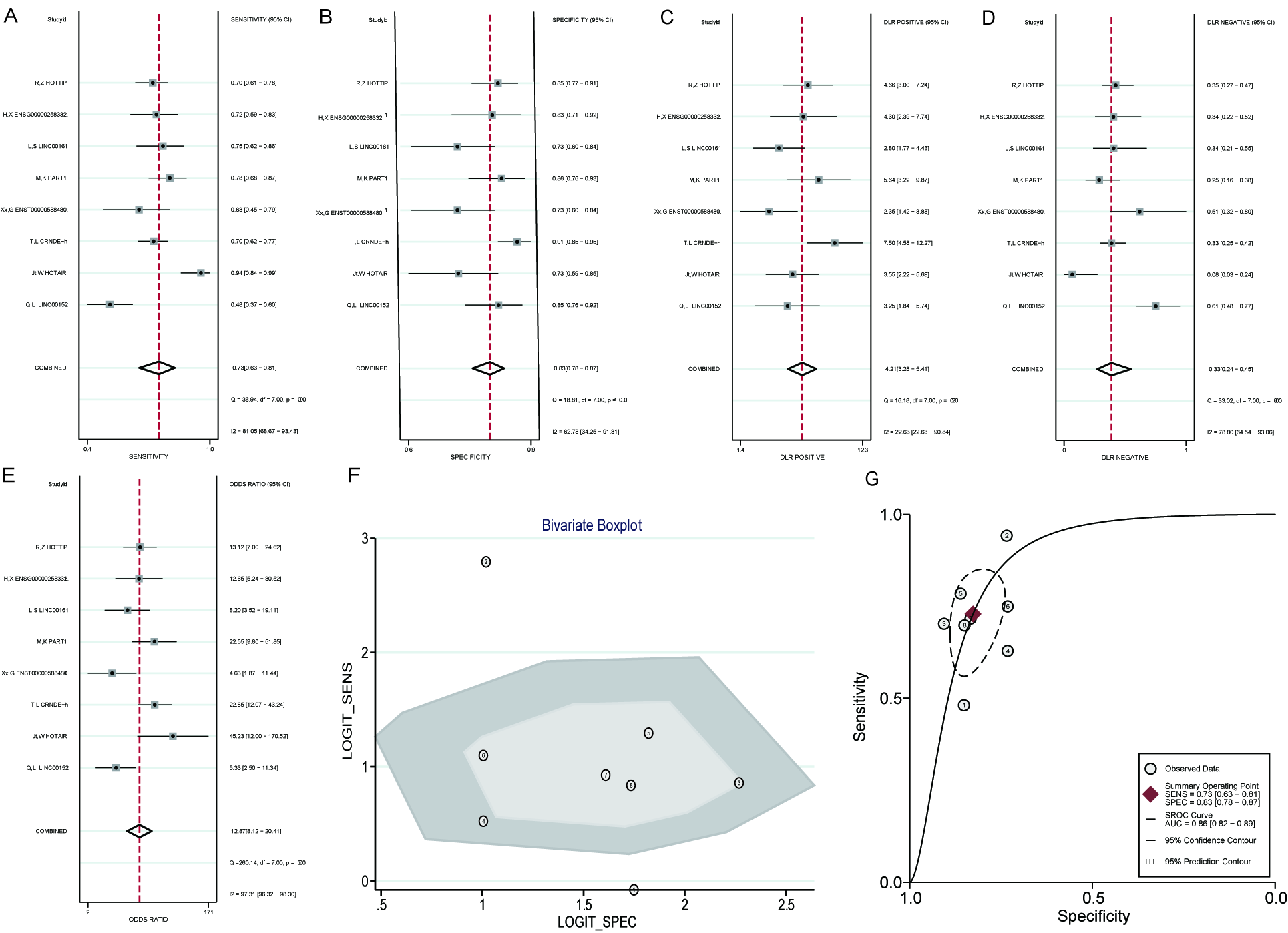

Supplement: Supplementary Materials — Figure S1: the study selection procedure for meta-analysis was conducted to the PRISMA statement. Supplementary 2. Table S1: PRISMA Checklist. Supplementary 3. Table S2: the inclusion and exclusion of two investigators. Supplementary 4. Figure S2: the Bivariate Boxplot of the 28 included study to assess the heterogeneity in the included studies. Supplementary 5. Figure S3: forest plot of sensitivity, specificity, DLR+, DLR-, DOR, and the Bivariate Boxplot an SROC of the liquid exosome lncRNA for the diagnosis of digestive system tumor among 8 studies. (A) Sensitivity, (B) specificity, (C) DLR+, (D) DLR-, (F) Bivariate Boxplot, (G) SROC. Supplementary 6. Figure S4: forest plot of sensitivity, specificity, DLR+, DLR-, DOR, and the Bivariate Boxplot an SROC of the liquid exosomes lncRNA for the diagnosis of urinary system tumor among 15 studies. (A) Sensitivity, (B) specificity, (C) DLR+, (D) DLR-, (F) Bivariate Boxplot, (G) SROC. Supplementary 7. Figure S5: forest plot of sensitivity, specificity, DLR+, DLR-, DOR, and the Bivariate Boxplot an SROC of the liquid exosomes lncRNA for the diagnosis in the group of blood sample type among 18 studies. (A) Sensitivity, (B) specificity, (C) DLR+, (D) DLR-, (F) Bivariate Boxplot, (G) SROC. Supplementary 8. Figure S6: forest plot of sensitivity, specificity, DLR+, DLR-, DOR, and the Bivariate Boxplot an SROC of the liquid exosome lncRNA for the diagnosis in the group of urine sample type among 10 studies. (A) Sensitivity, (B) specificity, (C) DLR+, (D) DLR-, (F) Bivariate Boxplot, (G) SROC. Supplementary 9. Figure S7: forest plot of sensitivity, specificity, DLR+, DLR-, DOR, and the Bivariate Boxplot an SROC of the liquid exosome lncRNA for the diagnosis in the group of sample size > 70 among 15 studies. (A) Sensitivity, (B) specificity, (C) DLR+, (D) DLR-, (F) Bivariate Boxplot, (G) SROC. Supplementary 10. Figure S8: forest plot of sensitivity, specificity, DLR+, DLR-, DOR, and the Bivariate Boxplot an SROC of the liquid exoso [file 6786875.f1.zip › Figure S4.tif]

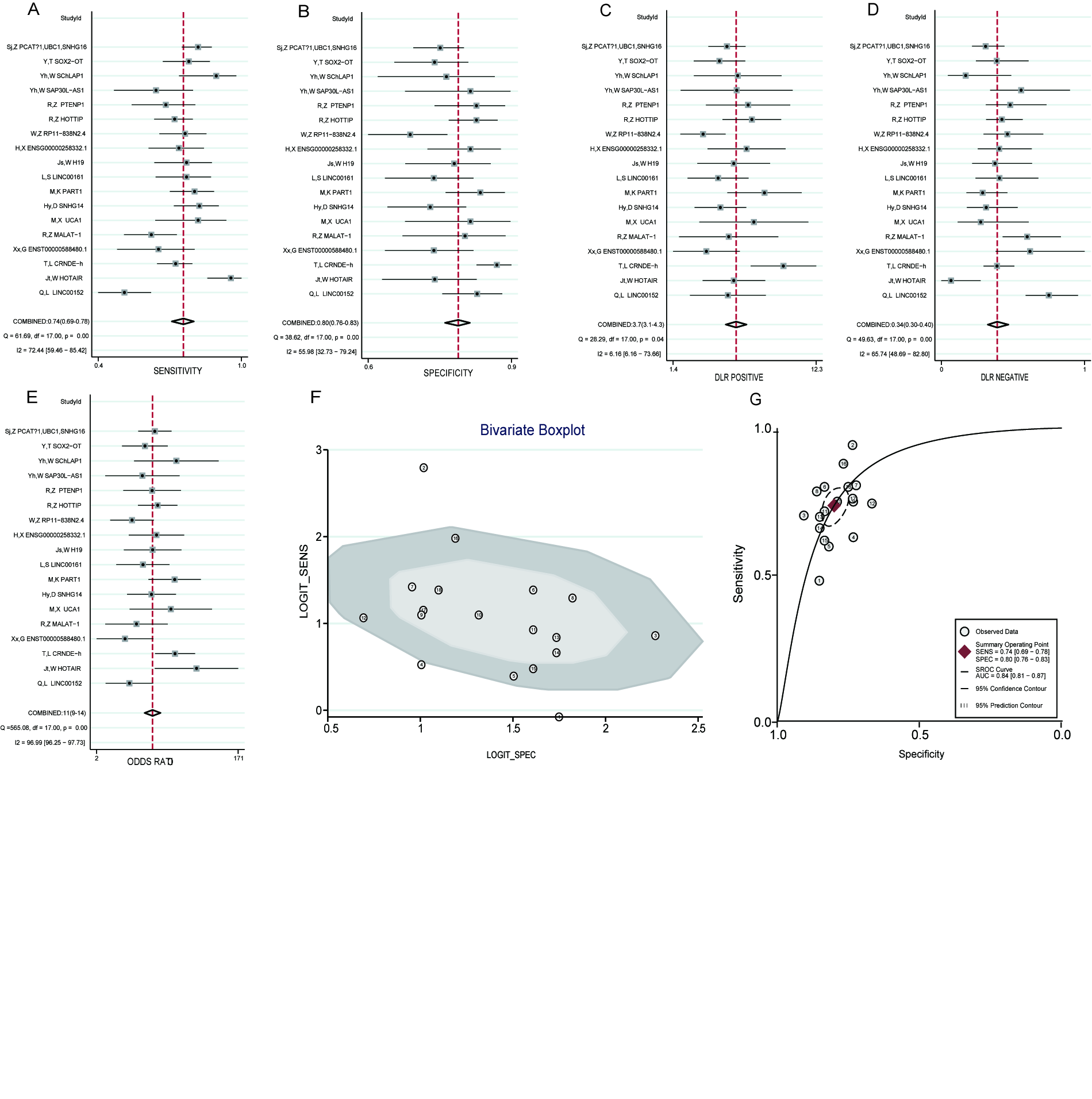

Supplement: Supplementary Materials — Figure S1: the study selection procedure for meta-analysis was conducted to the PRISMA statement. Supplementary 2. Table S1: PRISMA Checklist. Supplementary 3. Table S2: the inclusion and exclusion of two investigators. Supplementary 4. Figure S2: the Bivariate Boxplot of the 28 included study to assess the heterogeneity in the included studies. Supplementary 5. Figure S3: forest plot of sensitivity, specificity, DLR+, DLR-, DOR, and the Bivariate Boxplot an SROC of the liquid exosome lncRNA for the diagnosis of digestive system tumor among 8 studies. (A) Sensitivity, (B) specificity, (C) DLR+, (D) DLR-, (F) Bivariate Boxplot, (G) SROC. Supplementary 6. Figure S4: forest plot of sensitivity, specificity, DLR+, DLR-, DOR, and the Bivariate Boxplot an SROC of the liquid exosomes lncRNA for the diagnosis of urinary system tumor among 15 studies. (A) Sensitivity, (B) specificity, (C) DLR+, (D) DLR-, (F) Bivariate Boxplot, (G) SROC. Supplementary 7. Figure S5: forest plot of sensitivity, specificity, DLR+, DLR-, DOR, and the Bivariate Boxplot an SROC of the liquid exosomes lncRNA for the diagnosis in the group of blood sample type among 18 studies. (A) Sensitivity, (B) specificity, (C) DLR+, (D) DLR-, (F) Bivariate Boxplot, (G) SROC. Supplementary 8. Figure S6: forest plot of sensitivity, specificity, DLR+, DLR-, DOR, and the Bivariate Boxplot an SROC of the liquid exosome lncRNA for the diagnosis in the group of urine sample type among 10 studies. (A) Sensitivity, (B) specificity, (C) DLR+, (D) DLR-, (F) Bivariate Boxplot, (G) SROC. Supplementary 9. Figure S7: forest plot of sensitivity, specificity, DLR+, DLR-, DOR, and the Bivariate Boxplot an SROC of the liquid exosome lncRNA for the diagnosis in the group of sample size > 70 among 15 studies. (A) Sensitivity, (B) specificity, (C) DLR+, (D) DLR-, (F) Bivariate Boxplot, (G) SROC. Supplementary 10. Figure S8: forest plot of sensitivity, specificity, DLR+, DLR-, DOR, and the Bivariate Boxplot an SROC of the liquid exoso [file 6786875.f1.zip › Figure S5.tif]

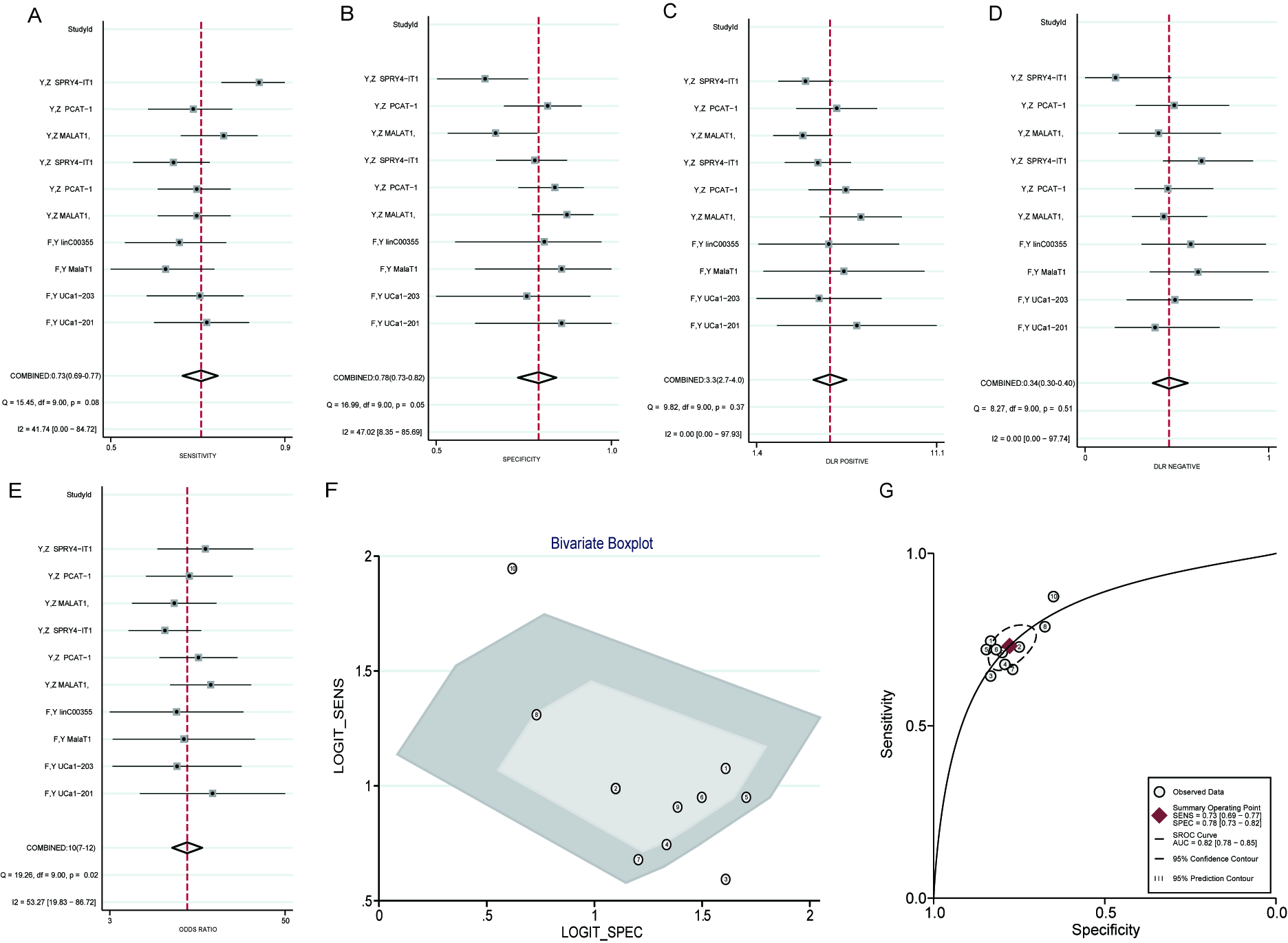

Supplement: Supplementary Materials — Figure S1: the study selection procedure for meta-analysis was conducted to the PRISMA statement. Supplementary 2. Table S1: PRISMA Checklist. Supplementary 3. Table S2: the inclusion and exclusion of two investigators. Supplementary 4. Figure S2: the Bivariate Boxplot of the 28 included study to assess the heterogeneity in the included studies. Supplementary 5. Figure S3: forest plot of sensitivity, specificity, DLR+, DLR-, DOR, and the Bivariate Boxplot an SROC of the liquid exosome lncRNA for the diagnosis of digestive system tumor among 8 studies. (A) Sensitivity, (B) specificity, (C) DLR+, (D) DLR-, (F) Bivariate Boxplot, (G) SROC. Supplementary 6. Figure S4: forest plot of sensitivity, specificity, DLR+, DLR-, DOR, and the Bivariate Boxplot an SROC of the liquid exosomes lncRNA for the diagnosis of urinary system tumor among 15 studies. (A) Sensitivity, (B) specificity, (C) DLR+, (D) DLR-, (F) Bivariate Boxplot, (G) SROC. Supplementary 7. Figure S5: forest plot of sensitivity, specificity, DLR+, DLR-, DOR, and the Bivariate Boxplot an SROC of the liquid exosomes lncRNA for the diagnosis in the group of blood sample type among 18 studies. (A) Sensitivity, (B) specificity, (C) DLR+, (D) DLR-, (F) Bivariate Boxplot, (G) SROC. Supplementary 8. Figure S6: forest plot of sensitivity, specificity, DLR+, DLR-, DOR, and the Bivariate Boxplot an SROC of the liquid exosome lncRNA for the diagnosis in the group of urine sample type among 10 studies. (A) Sensitivity, (B) specificity, (C) DLR+, (D) DLR-, (F) Bivariate Boxplot, (G) SROC. Supplementary 9. Figure S7: forest plot of sensitivity, specificity, DLR+, DLR-, DOR, and the Bivariate Boxplot an SROC of the liquid exosome lncRNA for the diagnosis in the group of sample size > 70 among 15 studies. (A) Sensitivity, (B) specificity, (C) DLR+, (D) DLR-, (F) Bivariate Boxplot, (G) SROC. Supplementary 10. Figure S8: forest plot of sensitivity, specificity, DLR+, DLR-, DOR, and the Bivariate Boxplot an SROC of the liquid exoso [file 6786875.f1.zip › Figure S6.tif]

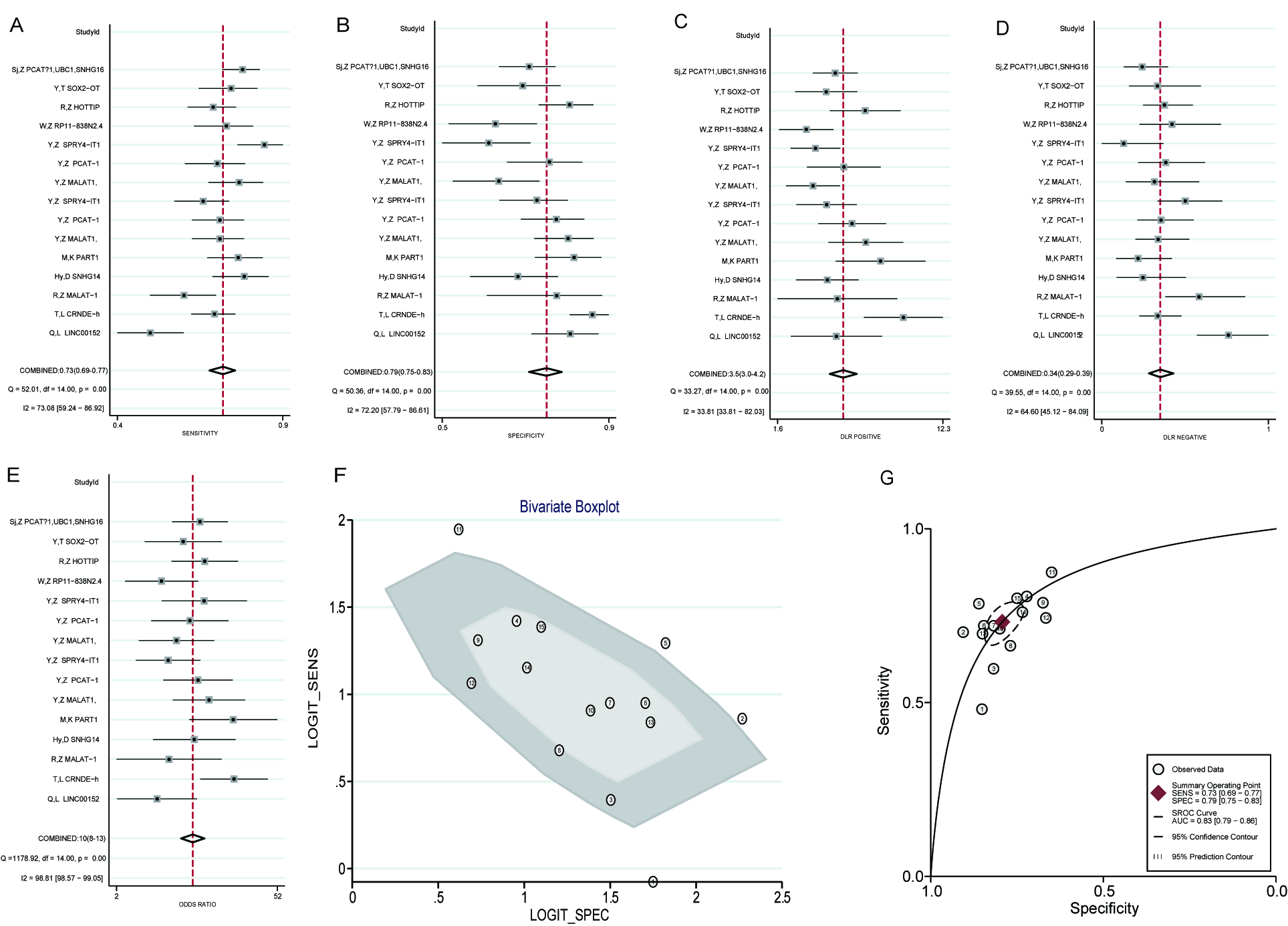

Supplement: Supplementary Materials — Figure S1: the study selection procedure for meta-analysis was conducted to the PRISMA statement. Supplementary 2. Table S1: PRISMA Checklist. Supplementary 3. Table S2: the inclusion and exclusion of two investigators. Supplementary 4. Figure S2: the Bivariate Boxplot of the 28 included study to assess the heterogeneity in the included studies. Supplementary 5. Figure S3: forest plot of sensitivity, specificity, DLR+, DLR-, DOR, and the Bivariate Boxplot an SROC of the liquid exosome lncRNA for the diagnosis of digestive system tumor among 8 studies. (A) Sensitivity, (B) specificity, (C) DLR+, (D) DLR-, (F) Bivariate Boxplot, (G) SROC. Supplementary 6. Figure S4: forest plot of sensitivity, specificity, DLR+, DLR-, DOR, and the Bivariate Boxplot an SROC of the liquid exosomes lncRNA for the diagnosis of urinary system tumor among 15 studies. (A) Sensitivity, (B) specificity, (C) DLR+, (D) DLR-, (F) Bivariate Boxplot, (G) SROC. Supplementary 7. Figure S5: forest plot of sensitivity, specificity, DLR+, DLR-, DOR, and the Bivariate Boxplot an SROC of the liquid exosomes lncRNA for the diagnosis in the group of blood sample type among 18 studies. (A) Sensitivity, (B) specificity, (C) DLR+, (D) DLR-, (F) Bivariate Boxplot, (G) SROC. Supplementary 8. Figure S6: forest plot of sensitivity, specificity, DLR+, DLR-, DOR, and the Bivariate Boxplot an SROC of the liquid exosome lncRNA for the diagnosis in the group of urine sample type among 10 studies. (A) Sensitivity, (B) specificity, (C) DLR+, (D) DLR-, (F) Bivariate Boxplot, (G) SROC. Supplementary 9. Figure S7: forest plot of sensitivity, specificity, DLR+, DLR-, DOR, and the Bivariate Boxplot an SROC of the liquid exosome lncRNA for the diagnosis in the group of sample size > 70 among 15 studies. (A) Sensitivity, (B) specificity, (C) DLR+, (D) DLR-, (F) Bivariate Boxplot, (G) SROC. Supplementary 10. Figure S8: forest plot of sensitivity, specificity, DLR+, DLR-, DOR, and the Bivariate Boxplot an SROC of the liquid exoso [file 6786875.f1.zip › Figure S7.tif]

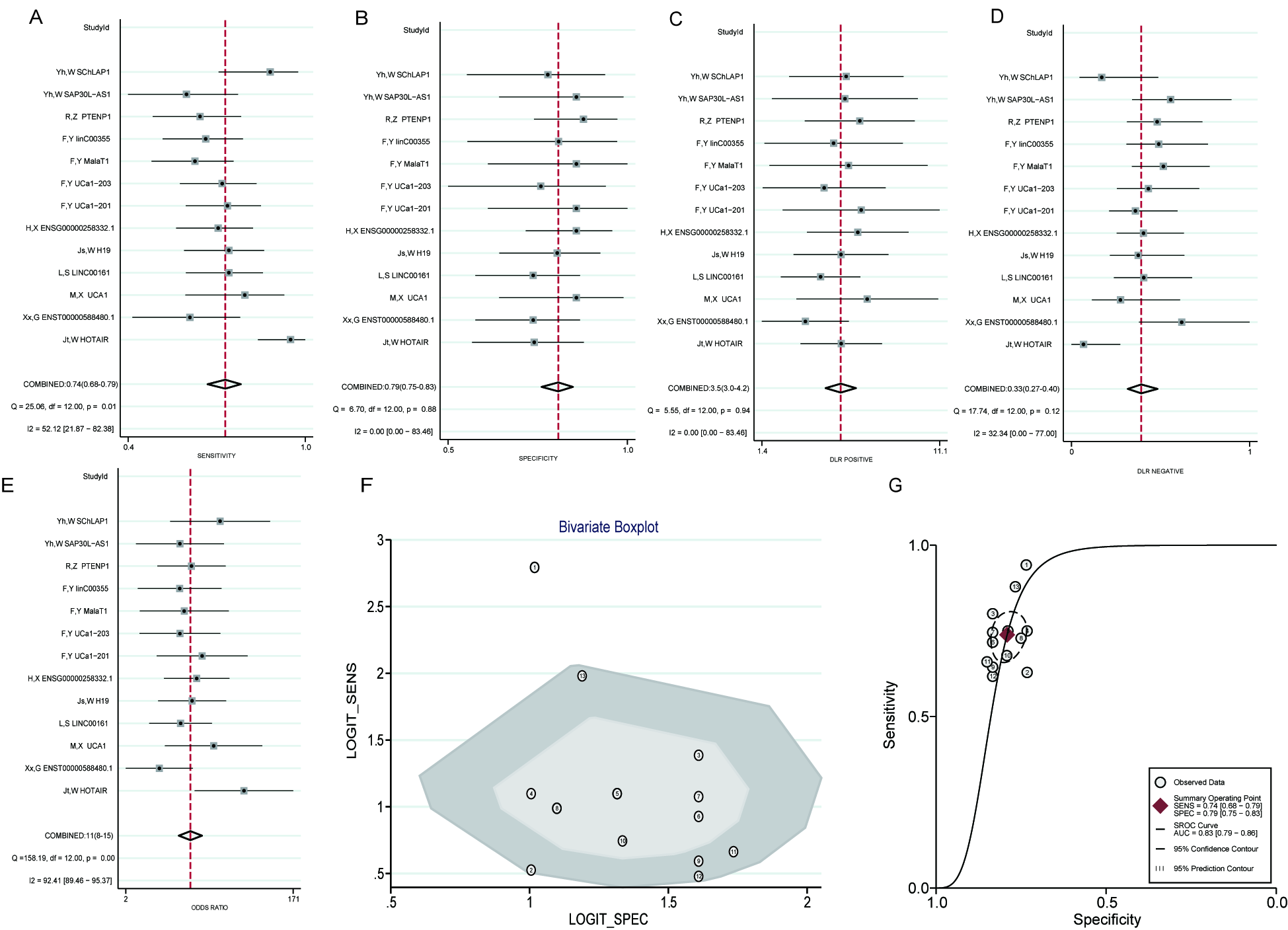

Supplement: Supplementary Materials — Figure S1: the study selection procedure for meta-analysis was conducted to the PRISMA statement. Supplementary 2. Table S1: PRISMA Checklist. Supplementary 3. Table S2: the inclusion and exclusion of two investigators. Supplementary 4. Figure S2: the Bivariate Boxplot of the 28 included study to assess the heterogeneity in the included studies. Supplementary 5. Figure S3: forest plot of sensitivity, specificity, DLR+, DLR-, DOR, and the Bivariate Boxplot an SROC of the liquid exosome lncRNA for the diagnosis of digestive system tumor among 8 studies. (A) Sensitivity, (B) specificity, (C) DLR+, (D) DLR-, (F) Bivariate Boxplot, (G) SROC. Supplementary 6. Figure S4: forest plot of sensitivity, specificity, DLR+, DLR-, DOR, and the Bivariate Boxplot an SROC of the liquid exosomes lncRNA for the diagnosis of urinary system tumor among 15 studies. (A) Sensitivity, (B) specificity, (C) DLR+, (D) DLR-, (F) Bivariate Boxplot, (G) SROC. Supplementary 7. Figure S5: forest plot of sensitivity, specificity, DLR+, DLR-, DOR, and the Bivariate Boxplot an SROC of the liquid exosomes lncRNA for the diagnosis in the group of blood sample type among 18 studies. (A) Sensitivity, (B) specificity, (C) DLR+, (D) DLR-, (F) Bivariate Boxplot, (G) SROC. Supplementary 8. Figure S6: forest plot of sensitivity, specificity, DLR+, DLR-, DOR, and the Bivariate Boxplot an SROC of the liquid exosome lncRNA for the diagnosis in the group of urine sample type among 10 studies. (A) Sensitivity, (B) specificity, (C) DLR+, (D) DLR-, (F) Bivariate Boxplot, (G) SROC. Supplementary 9. Figure S7: forest plot of sensitivity, specificity, DLR+, DLR-, DOR, and the Bivariate Boxplot an SROC of the liquid exosome lncRNA for the diagnosis in the group of sample size > 70 among 15 studies. (A) Sensitivity, (B) specificity, (C) DLR+, (D) DLR-, (F) Bivariate Boxplot, (G) SROC. Supplementary 10. Figure S8: forest plot of sensitivity, specificity, DLR+, DLR-, DOR, and the Bivariate Boxplot an SROC of the liquid exoso [file 6786875.f1.zip › Figure S8.tif]
